# A Perspective on COVID-19 Management

**DOI:** 10.3390/jcm10081586

**Published:** 2021-04-09

**Authors:** Krešimir Pavelić, Sandra Kraljević Pavelić, Bianca Brix, Nandu Goswami

**Affiliations:** 1 Faculty of Medicine, Juraj Dobrila University of Pula, Zagrebačka 30, 52100 Pula, Croatia; pavelic@unipu.hr; 2 Faculty of Health Studies, University of Rijeka, Viktora Cara Emina 5, 51000 Rijeka, Croatia; sandrakp@biotech.uniri.hr; 3 Physiology Division, Otto Loewi Research Center for Vascular Biology, Immunology and Inflammation, Medical University of Graz, Neue Stiftingtalstraße 6/D.05, 8010 Graz, Austria; bianca.brix@medunigraz.at

**Keywords:** SARS-CoV-2, endothelium, COVID-19 management

## Abstract

A novel coronavirus—Severe Acute Respiratory Syndrome-Coronavirus 2 (SARS-CoV-2)—outbreak correlated with the global coronavirus disease 2019 (COVID-19) pandemic was declared by the WHO in March 2020, resulting in numerous counted cases attributed to SARS-CoV-2 worldwide. Herein, we discuss current knowledge on the available therapy options for patients diagnosed with COVID-19. Based on available scientific data, we present an overview of solutions in COVID-19 management by use of drugs, vaccines and antibodies. Many questions with non-conclusive answers on the measures for the management of the COVID-19 pandemic and its impact on health still exist—i.e., the actual infection percentage of the population, updated precise mortality data, variability in response to infection by the population, the nature of immunity and its duration, vaccine development issues, a fear that science might end up with excessive promises in response to COVID-19—and were raised among scientists. Indeed, science may or may not deliver results in real time. In the presented paper we discuss some consequences of disease, its detection and serological tests, some solutions to disease prevention and management, pitfalls and obstacles, including vaccination. The presented ideas and data herein are meant to contribute to the ongoing debate on COVID-19 without pre-selection of available information.

## 1. Introduction

In December 2019, the new strain of the coronavirus captured attention first in Wuhan and then globally. Soon, the World Health Organization (WHO) declared a pandemic in March 2020. Viral infections with SARS-CoV-2 have challenged almost every country worldwide in terms of health system capacities and economic burden. Some open questions then arose and require scientifically-based answers such as those on the origin and cause of the disease, actual infection rate among the population, updated mortality data, variability in response to the infection by the population, the nature of immunity and immunity duration, the usefulness of the vaccine approach in disease management and the relevance of virus mutations. In addition to these questions, a number of other, non-medical issues related to the social-humanistic field, that we will not discuss herein, have been also raised in public. Just to mention one of them, an extreme choice pushed governments to balance between population health preservation and preservation of the society functioning and economy. Indeed, the situation that is now in place upon proclamation of the SARS-CoV-2 pandemic has put humanity in a huge dilemma—to undertake massive measures directed towards health of the population in relation to one disease or to preserve social and economic well-being. A “lockdown” as it was imposed to the society, has a huge impact, both economically and socially. All of us humans are meant to work, interact, earn, live and socialize, thus creating the society through social interactions. Observed damage occurred due to drastic measures established globally to counteract one particular disease, which was not confined to one particular societal sector. Some authors have consequently suggested a novel policy directed to COVID-19 innovation investments as a possible solution [1]. In addition, the number of published papers and research in general on the coronavirus has simply exploded recently contributing to expanded knowledge on the clinical presentation of COVID-19, which is essential in shaping appropriate medical solutions to the current situation. Against the background of this landscape, we will discuss here the characteristics of the SARS-CoV-2 correlated with the COVID-19 disease as published in the literature, its testing and therapy as well as the vaccine development status. Other open questions such as comprehensive epidemiologic analyses are not covered in this review.

## 2. Characteristics of SARS-CoV-2

The origin of SARS-CoV-2 is still controversial. Two hypothesis, one based on the zoonotic transfer to humans and the second covering the probability of a hybrid virus construction and escape from the lab, may be substantiated by the literature and/or other available information [2,3]. Very briefly, SARS-CoV-2 is classified within the family *Coronaviridae*, genus *Betacoronavirus* with 14 open reading frames (ORFs) encoding for 27 proteins. Its important feature is the spike glycoprotein (S) required for the virus binding to the host cell receptor ACE2. The S protein has the S1 domain, responsible for the receptor binding and the host virus range, while the S2 domain is responsible for cell membrane fusion. Specifically, SARS-CoV-2 has a receptor binding domain (RBD) within the S1 subunit that binds to ACE2 with a high affinity and is the main S1 subunit component that drives the SARS-CoV-2 binding to ACE2 [4]. Other SARS-CoV-2 structural proteins are the small envelope protein (E), matrix protein (M) and nucleocapsid protein (N) [5]. A cleavage domain in the S protein of SARS-CoV-2, named furin-cleavage domain, has not been previously reported in other SARS-CoV viruses. This S glycoprotein is cleaved by the host cell furin-like protease into S1 and S2 subunits [6] (Table 1). This SARS-CoV-2 spike protein has been suggested to be essential for the high transmissibility and infectivity of the virus observed at the beginning of the pandemic proclamation [7,8]. Currently, identified variants from Brazil, the United Kingdom and South Africa (see the subchapter below) have substantially higher binding affinity to ACE2 due to RBD mutations which confer to their increased transmissibility [9].

Some papers discuss a possible linkage of COVID-19 to coronaviruses, especially to bat coronaviruses where robust data is presented on the similarities between the genetic material of SARS-CoV-2 and other SARS-CoV viruses [10]. The ubiquitous expression of furin in different organs and tissues has also been suggested to confer SARS-CoV-2 with the ability to infect body parts insensitive to other CoVs, leading to a systemic infection in the body [11]. However, no unambiguous proof of the natural zoonotic transfer origin or synthetic origin has been provided so far. Published scientific literature mainly elaborates the evolutionary aspects and natural origin of the SARS-CoV-2. Still, the need for an open debate on the SARS-CoV-2 origin has a wider implication for society as it is known that scientists already worked on the production of recombinant SARS-CoV viruses before the year 2020 [12]. Many groups worked, for example, on the isolation and propagation of SARS-CoV viruses in the lab that may give rise to mutated virus types as well [13]. Still, a large number of scientists support the hypothesis of the virus passing from animals to humans, probably from bats. Such explanation is the most elegant. For example, some reports on different coronaviruses identified in bats were published in China. The presented results were obtained from bat fecal swabs collected in an abandoned mineshaft in Mojiang County, Yunnan Province, China back in 2016 [14] and 2017 [15]. Recent efforts were focused on studying the molecular characteristics of the SARS-CoV-2 virus, including its newly published genome sequence compared to these evolutionary neighbors, as i.e., identified in bats. Very importantly, in these discussions almost no attention was given to the physical origins (whole viral particles studies) of those close genetic relatives and SARS-CoV-2 presumed ancestors. Emphasis is now put on two of those viral genetic sequences, namely BtCoV/4991 and RaTG13 which were reported in the literature, as collected from the mine shaft in Yunnan Province, China by researchers from the Zheng-li Shi Laboratory at the Wuhan Institute of Virology. In the published papers from this group however, the sequence under the name RaTG13 is not explicitly stated [13,14,16]. In the paper of Zeng et al., three novel SARS-CoVs were identified to be able to use human angiotensin converting enzyme 2 (ACE2) as a cellular entry receptor due to their S glycoprotein properties. Those were tested on human HeLa cells (human cervical carcinoma cell line) to study virus infectivity. The virus replicated efficiently in the human ACE2-expressing cells [16]. The explicit report of the RATG13 viral sequence was published in scientific reports from 2020 for the bat species *Rhinolophus affinis*, where a 96.2% homology to the SARS-CoV-2 genetic sequence has been emphasized [17], and *Rhinolophus malayanus* with the viral sequence identified and designated as RmYNO2 showing a 93% homology to SARS-CoV-2 [18]. Some scientists demand for more research and further validation [19]. 

Both the “natural zoonotic transfer” or “lab escape” theories might partially explain the origin of the polybase cleavage site of furin, which is the area of the S glycoprotein that makes it susceptible to cleavage by the host enzyme furin and which greatly promotes the spread of the virus in the body. This novel furin place in SARS-CoV-2 is relevant in the infection process of humans, indeed distinguishing it from its closest relatives [7,20,21]. This explains the extreme affinity of the S glycoprotein virus for human receptors, which surprised virologists, also due to the SARS-CoV-2 unique adaptation to infect humans [22,23]. In summary, it is clear that science cannot yet give a conclusive answer to the question of the SARS-CoV-2 origin.

**Table 1:** Key differences between SARS-CoV-2 and its closest suggested relative RaTG13.

Feature of SARS-CoV-2 Genetic Material	Reference
A cleavage site in the spike protein (glycoprotein S) of SARS-CoV-2 activated by the host-cell enzyme furin, previously not identified in other beta-CoVs betacoronaviruses belonging to lineage b Genetic sequence: CCT CGG CGG GCA Corresponding aminoacid sequence: PRRA (Pro-Arg-Arg-Ala)	Zhang et al., 2020 [24] Coutard et al., 2020 [7]
SARS-CoV-2 four amino acids that insert PRRA into the furin cleavage site were reported not to be in frame with the rest of sequence, when compared with the MP789 and the RaTG13 sequences, and contain a *Fau*I enzyme restriction site that could allow use of recombinant genetic methods.	Segreto et al., 2020 [25]

### SARS-CoV-2 New Variants

Recent sequencing data show diverse SARS-Cov-2 sequence variants circulating globally. A Brazilian SARS-Cov-2 lineage B.1.1.28 known as P.1 (501Y.V3) has been for example, spreading and importing to other countries since February 2020 [26]. Besides, other studied variants include the SARS-Cov-2 lineage B.1.1.7 (501Y.V1) reported in the UK and other European countries and a variant from South Africa B.1.351 (501Y.V2) probably originating from the B.1.1.28 lineage. Both the B1.1.28. and B.1.351 display the mutation E484K relevant for the S protein activity [26]. According to the published data, the lineage B.1.1.7 accumulated 17 lineage-defining mutations as part of previous evolution and at the end of 2020 it accounted for 28% of SARS-CoV-2 infection in the UK [27]. 

Centers for Disease Control and Prevention (CDC) reports that the relevant mutation of this variant is identified in the receptor binding domain (RBD) of the S protein at position 501 but other mutations include 69/70 deletion in the S protein and P681H near the S1/S2 furin cleavage site. A mutation means that an actual change in sequence occurred. The viral genomes that accordingly differ in sequence are referred to as variants. A variant is a strain if a different phenotype occurred, such as for example increased or decreased transmissibility or virulence. The arising of new SARS-CoV-2 variants is an expected phenomenon. Indeed, viruses naturally mutate over time and adapt to the environmental conditions. Very specific and new massive epidemiologic measures without precedence directed to control the SARS-CoV-2 spread have been implemented worldwide including lockdown measures, distancing, travel bans and mask wearing. Epidemiologists consequently discussed the effects of such measures on the SARS-CoV-2 evolution. For example, on one side, some ask for even stricter measures due to new variants [27], while others ask for immediate abandoning of such measures as the possible immune escape due to the selection pressure from both measures and vaccines might create ever more vaccine resistant, and potentially dangerous, virus variants [28]. Scientific data on the exact outcomes of the observed genetic variants of SARS-CoV-2 in terms of infectivity and morbidity is inconclusive. Nevertheless, it seems that these variants may still be well recognized by the immune system due to small sequence changes and probably do not confer higher mortality or to specific clinically relevant outcomes. It should be also emphasized that RNA viruses have properties that underlie all these observations. For example, RNA viruses have generally a large population size, their propulsive replication is driven by new mutants with increased fitness and RNA viruses have a high mutation rate [29]. SARS-CoV-2 is not an exception and since the virus was reported for the first time in China, different mutant sequences have been detected [26]. We can accordingly expect further evolution of the SARS-CoV-2 that will be interconnected with a number of factors, including the type of measures applied in the future. It is still not possible to predict whether this will lead to changes in the phenotype and new strains.

## 3. Is COVID-19 a “Bad Endothelium” Disease?

Clinical presentation of COVID-19 includes fever, cough, dyspnea, abdominal pain and diarrhea [30,31]. About 15% of patients affected by COVID-19 need to be hospitalized and 5% of patients are critically ill, develop acute respiratory distress and need to be admitted to intensive care units [32]. Acute respiratory distress syndrome (ARDS) is one of the highly relevant manifestations in COVID-19 patients. According to Gattinoni et al. (2020) [33] the term “atypical ARDS” may be used to describe specific clinical, mechanical and radiological criteria observed in some COVID-19 patients. These criteria are not merely based on the severity of gas exchange and accordingly, the management of individual patients needs to take into consideration various factors, not just gas exchange that defines the general ARDS [33]. Current evidence indicates that the risk of severe forms of the disease increases with age, male sex, and with co-morbidities such as chronic lung disease, cardiovascular disease and diabetes [34,35,36,37,38,39]. COVID-19 potentially affects the nervous system leading to a sudden loss of smell or taste sensation [40,41,42,43]. There is a general consensus that vascular endothelial function can be regarded as a marker of the net harmful effects of cardiovascular risk factors on the vascular wall [44,45,46]. As COVID-19 syndrome is associated with multisystem inflammation [47], the pattern of organ damage caused by COVID-19 occurring in patients with COVID-19 is still incompletely understood, current treatment options are limited and improved understanding of the risk for severe and fatal COVID-19 outcomes is urgently needed [48]. Patients with severe COVID-19 can develop COVID-19-associated coagulopathy, with features of both disseminated intravascular coagulation and thrombotic microangiopathy, resulting in widespread microvascular thrombosis that may involve consumption of coagulation factors [49] and the liver [50]. This appears to have a causal relationship with the inflammatory and reparative processes involving diffuse alveolar damage (DAD), because thrombi are frequently detected in small pulmonary arteries, most likely secondary to endothelial damage [51]. The endothelial damage could occur due to the direct viral infection of the endothelial cells, which express ACE-2 receptors, or to a host response [52]. Furthermore, the alveolar fibrin deposition in DAD may affect the delicate local balance of fibrinolysis and coagulation [52]. A combination of alveolar and endothelial damage of smaller vessels may be followed by microvascular pulmonary thrombosis, which could then extend to larger vessels. Additionally, elevated D-dimer has been seen in patients with COVID-19, especially those at a severe stage [31,50]. It is well known that elevated D-dimer concentrations are associated with acute pulmonary emboli (APE), deep venous thrombosis (DVT), cancer, peripheral vascular disease, inflammatory diseases and pregnancy. Patients with COVID-19, including those not on respirators but confined to bed, develop DVT and APE [53] much earlier than expected [52]. Despite the usage of prophylactic anticoagulation, autopsy reports have shown that deaths in COVID-19 may be caused by the thrombosis in segmental and subsegmental pulmonary arterial vessels [50].

## 4. Detection of SARS-CoV-2 and Serological Tests

The currently used standard method for diagnosis of SARS-CoV-2 infections is the genetic test by real-time polymerase chain reaction (RT-PCR). A complete diagnostic procedure should be however, performed additionally to clinically confirm the infection. Testing of the healthy population or people without symptoms may only be used, with a very high degree of caution, in an eventual epidemiologic monitoring. Current testing and diagnostic procedures have been found to yield both false negative and false positive test results, both leading to unwanted virus spreading possibilities, misleading information and/or unnecessary stress to involved individuals [54,55]. The options and pitfalls of the current limitations of the methods for detecting SARS-CoV-2 have been recently summarized in Mathuria et al. [56]. They also emphasize the need of more accurate and reliable diagnostic tests for SARS-CoV-2 infection. Besides RT-PCR, those may include immunological diagnostic tests as well. Particularly, serological tests may measure the response of antibodies to induced SARS-CoV-2 infections, which is important for studying the overall population’s immunity to SARS-CoV-2 [57]. Serological tests are a crucial tool in the treatment of infectious diseases, in the measurement of protective antibody titers upon vaccination, as well as in the assessment of the seroprevalence of immunity in the population. Currently, serological tests for SARS-CoV-2 include automated enzyme-linked immunosorbent assays (ELISA) or chemiluminescence enzyme immunoassays (CLIA) as well as rapid detection lateral flow immunoassays (LFIA) [58]. ELISA and LFIA rely on the use of recombinant antigens such as the S glycoprotein. The receptor-binding domain (RBD), which is part of the S glycoprotein or viral nucleoproteins, is also used [59]. Still, all these tests have shown different performance rates depending on the clinical course and the test procedure [60,61]. Even though it is a useful addition to other diagnostic methods, serological tests fail to answer the question on the different antibody responses in patients with severe symptoms in comparison with those showing mild or no symptoms at all. It is unknown whether the presence of the antibody binding to spikes (S glycoproteins) or the corresponding S glycoprotein receptor binding domain (RBD) antigens correlate with viral neutralization. It is assumed that a person with antibodies to SARS-CoV-2 will be less susceptible to re-infection, and that these antibodies will contribute to a reduction of disease severity or limit the spread of the virus. Some (or even the majority) of patients may indeed develop a robust antibody response. These people can return to normal life and work without special measures. Detection of a protective immune response is also important for healthcare professionals that are in contact with patients on a daily basis. Furthermore, people with confirmed immunity to SARS-CoV-2 might well be spared from quarantine and social distancing, both measures that abrogate societal function at its deepest level. This is why more research into the duration and the immune response length and quality to SARS-CoV-2 infection is required. Only comprehensive data will allow researchers to draw correlations between the immune response and protection from re-infection [62]. 

## 5. Strategies for Management of COVID-19: Current Status, Pitfalls and Obstacles

In the scientific community, research related to any novel disease mainly goes in two directions. One direction is the investigation and usage of effective treatment(s) with potential antiviral drugs or antibodies, and the second one is the finding of adequate approaches to strengthen specific immunity by vaccination.

### 5.1. Clinical Trials of Potential Antiviral Drugs

A number of clinical trials with existing drugs repurposed towards SARS-CoV-2 treatment have been established promptly. The results were published in the scientific literature. Here it should be noted that the history of antiviral drugs research, with some exceptions, has been often marked by failures. When it comes to the investigation of potential drugs in COVID-19 treatment, non-conclusive results are based partially on the early stages of testing and are sometimes even negative. For example, lopinavir–ritonavir combination showed no relevant benefits in severe COVID-19 patients [63]. Still, some authors point out the numerically lower mortality rate and lower intensive care unit stays of patients treated with lopinovir–ritonavir in this study and, therefore, suggest continuation of the trials and re-analysis of the results. In addition, WHO launched the initiative SOLIDARITY, aimed at monitoring COVID-19 patients globally, randomized to local standard care or one of the four drug regimens: antiviral drug remdesivir, the malaria medication chloroquine or hydroxychloroquine, a combination of HIV drugs lopinavir and ritonavir, and that combination plus interferon-beta [64]. Due to the high urgency associated with finding solutions for COVID-19 patients, clinical trials aiming to study the effects of drugs in COVID-19 patients are often performed on a trial-and-error basis. For example, safety considerations have been raised for usage of chloroquine and hydroxychloroquine [65]. On the other side, the combination of antiviral drugs and interferon beta showed no serious side effects [66]. The effects of corticosteroids (i.e., clinical trial registered under NCT04273321) and baricitinib (i.e., clinical trial registered under NCT04401579), which are normally used to treat rheumatoid arthritis, and camostat mesilate (i.e., clinical trial registered under NCT04353284), are also being tested. Other antiviral drugs, including favipiravir [67] and HIV antiretroviral drugs darunavir and cobicistat, are being studied in a clinical trial in China (clinical trial registered under NCT04252274). Data showing effectiveness in COVID-19 treatment were obtained also for the antiviral drug remdesivir [68]. The rationale for studying ramdesivir in COVID-19 patients can be explained by the characteristics of SARS-CoV-2 itself. The SARS-CoV-2 genomic material firstly translates from ORF1a and ORF1b to produce two large overlapping polyproteins, pp1a and pp1ab. These polyproteins are supplemented by protease enzymes encoded in nsp3 and nsp5. Subsequently, cleavage occurs between pp1a and pp1ab into nonstructural proteins 1–11 and 1–16, respectively [6,21,69]. They combine to accelerate the replication and transcription and probably do so with the help of nsp7 and nsp8 [70]. According to Gao et al. the RNA-dependent RNA polymerase (RdRp), named nsp12, is central to the coronavirus transcription machinery, and a target for the antiviral drug remdesivir. This polymerase has a conserved architecture and possesses a newly identified β-hairpin domain at its N terminus. A comparative analysis model shows how remdesivir binds to this polymerase [71]. Moreover, the anti-inflammatory medicine dexamethasone showed potential in COVID-19 patients receiving either invasive mechanical ventilation or oxygen alone [72]. Possible use of dexamethasone has also been recently suggested as a possible option in severe intubated patients at a dose of 6 mg once daily for up to 10 days [73] or in combination with nebulized triamcinolone for lung localization of the drug followed by administration of natural flavonoid luteolin because of its antiviral and anti-inflammatory properties aimed to reduce cytokine production in the lungs [74]. A recently published paper showed efficacy of high dosages of calcifediol, the 25-hydroxyvitamin D, in reduction of the number of hospitalized COVID-19 patients requiring intensive care unit. In this study, all patients received the same standard care based on the combination of hydroxychloroquine and azithromycin [75]. Finally, another drug—Ivermectin, an approved anti-parasitic drug—showed to be a promising candidate in early treatment of COVID-19 patients. The data was presented by the FLCCC Alliance [76] and is based on published studies showing its potential on SARS-CoV-2 inhibition [77].

### 5.2. Antibody Driven Treatment

Researchers are also trying to study the effects of antibody-rich plasma of COVID-19 recovered patients (known as “convalescent“ plasma) as a way of treatment or for boosting of immunity in patients recovering from COVID-19 [78,79]. One of the aims of plasma studies and antibody research is the identification of exactly one or more neutralizing antibodies that may disable infectious SARS-CoV-2, i.e., that are able to bind to the S glycoprotein and thus prevent entry into human cells. The immune response of B-cells would be then obtained. Still, the exact outcomes of this approach have not been rigorously measured and need to be more extensively researched [80]. The anti-virus antibodies may be evaluated for their effectiveness in COVID-19 treatment with a potential to counteract the SARS-CoV-2 infection. Which antibodies are the most effective ones however, still remains unknown: e.g., will we need a combination of antibodies or will one antibody suffice for efficient SARS-CoV-2 counteraction? It should be noted that monoclonal antibodies (mAbs) against tumors and autoimmune diseases are a huge business today although there are still relatively few antibodies on the market. The effectiveness of the antibody cocktail strategy has also been tested, for example, against Ebola [81]. Therefore, a similar scenario might be envisaged for COVID-19 even though antibody production is neither easy nor cheap. It is assumed that in the next five years it may be the main tool against the pandemic. For example, a recent study reports a possible mAb candidate by use of the plaque reduction neutralization test on VeroE6 cells infected with the pathogenic SARS-CoV-2. The authors prepared and tested neutralizing antibodies that targeted four distinct epitopes on the spike RBD of the SARS-CoV-2. They suggest that some of the tested antibodies (with the best performance) might be useful in the treatment of COVID-19 patients or for prophylactic immunization [82,83,84]. An interesting study on antibodies targeting SARS-CoV-2 has also been recently discussed in the scientific community. It was indeed shown that naturally formed neutralizing antibodies are able to target SARS-CoV-2 in humans, which was detected before the onset of the COVID-19 pandemic in people with SARS back in 2003. This antibody has the capability to target the SARS-CoV-2 S protein via the recognition of a highly conserved epitope in the S glycoprotein domain [85]. The unanswered question is which of the many monoclonal antibodies identified so far with a potential against SARS-CoV-2 is the best and why? The answer will not be straightforward as we do not really know the role of neutralizing antibodies in this disease [86]. Just a positive example: Wu et al. showed that a noncompeting pair of human neutralizing antibodies block COVID-19 virus binding to the receptor ACE2, which is essential for its entrance into the host cell [87]. The antibodies block the binding domain of the virus and the cellular receptor ACE2. The antibodies were able to reduce the virus titer in infected mouse lungs. These findings may emphasize the potential of COVID-19 antibody therapy. In Table 2 we present currently available data on therapy options for COVID-19 patients.

### 5.3. Vaccination

Finally, vaccination is pushed as one of the solutions to pandemics. Although T-cells are the most effective immune defence, their role in the fight against COVID-19 is currently not completely elucidated. Some people who have never been infected with SARS-CoV-2 possess a cellular response to that virus, most likely because they have been previously infected with other coronaviruses circulating in the population for years, probably those that cause frequent flu. However, such adaptive immune response of adequate durability and magnitude towards SARS-CoV-2 may fail to develop in some instances. Vaccines under development are thus mainly focusing on stimulating the host immune response, particularly to the S glycoprotein of SARS-CoV-2. Recently, it has been reported that SARS-CoV-2 virus-specific T-cells were detected in the majority of COVID-19 patients, specifically the circulating SARS-CoV-2-specific CD8+ and CD4+ T cells in about 70% and 100% of COVID-19 convalescent patients, respectively [116]. Of course, this does not necessarily mean that people who recovered from SARS-CoV-2 are safe from reinfection. In addition, immune evasion by SARS-CoV-2 may occur, e.g., in elderly patients, where a late T-cell response may be even detrimental to patients as it leads to inflammatory complications due to sustained high viral loads in the lungs. According to Giamarellos-Bourboulis et al. such immune dysregulation including cytokine production and hyper-inflammation in severe COVID-19 patients is underlined by IL-6-mediated low HLA-DR expression and lymphopenia [117]. At last, more data on SARS-CoV-2 proteins and epitopes that are recognized by T-cells is necessary for a proper vaccine hypothesis development. A recent paper was therefore aimed to evaluate the CD4+ and CD8+ T-cells responses in COVID-19 cases. The authors documented SARS-CoV-2 specific CD4+ T-cell and antibody responses in all analyzed COVID-19 cases. In addition, a very interesting finding of the authors was on pre-existing SARS-CoV-2-cross-reactive T-cell responses in healthy donors. They suggest an existence of a pre-existing immunity to SARS-CoV-2 in the population. Distinct specificity patterns between COVID-19 cases and unexposed healthy controls were shown as well [116]. The cross-reactivity was also documented in the study of Braun et al., where as many as 35% of host helper T-cells of unexposed healthy donors were cross-reactive to SARS-CoV-2 virus. The authors also identified helper T-cells targeting spikes in 15 of 18 patients hospitalized with SARS-Cov-2 [118]. It is plausible to assume therefore, that a large number of people can cope with the SARS-CoV-2 virus as they possess some residual immunity after exposure to similar viruses in the past and may not even need a vaccine.

In vaccine development, the final success depends on the following factors: infection end-point, transmission end-point and disease severity. Main technologies used currently in SARS-CoV-2 vaccine development at the moment include attenuated and inactivated virus vaccines, subunit protein vaccines, viral vector vaccines, nucleic acid vaccines and BCG vaccines [119].

According to the official NIH site accessed on 1 April 2021, 532 recruiting or non-recruiting clinical studies in phases I to III with different vaccines directed to control the COVID-19 are listed. Five vaccines have been approved so far for full use under emergency use authorization (BNT162b2 vaccine from Pfizer and the German company BioNTech, the mRNA-1273 vaccine from Moderna, Johnson & Johnson JNJ-78436735, AstraZeneca from University of Oxford and the British–Swedish company AstraZeneca, Sputnik V vaccine from the Gamaleya Research Institute, part of Russia’s Ministry of Health and Sinopharm from the China National Pharmaceutical Group) and one vaccine (Australia’s University of Queensland) was abandoned after trials. The vaccines are in their early application phases, have been developed in a rather short time-frame and their efficacy and safety profiles remain to be evaluated. For example, the most commonly reported side effects documented for the Pfizer vaccine by the vaccine producer include injection site reaction (pain, redness, warmth, mild swelling or firmness), fatigue, headache, muscle pain, chills, joint pain and fever. Other reported side effects include lymphadenopathy, Bell’s palsy or a condition that causes temporary facial paralysis, severe allergic reactions, appendicitis, acute myocardial infarction, and cerebrovascular accident [120]. The Advisory Committee on Immunization Practices (ACIP) COVID-19 at the CDC site reports six cases of anaphylaxis and 3150 health impact events defined as “unable to perform normal daily activities, unable to work, required care from doctor or health care professional” [121].

## 6. Conclusions

Excessive promises in response to COVID-19 research are expected from the scientific community. In fact, COVID-19 disease severity, in particular the mortality rate and infectivity may be calculated precisely only with a time-delay and a careful, very precise monitoring. Having in mind that so many open questions and lack of robust knowledge on SARS-CoV-2 and COVID-19 still exist, the comprehensive, methodologically correct and responsible approach of professionals and scientists towards clinical studies, research and management of this disease are required. Science should not be expected to provide finite answers as it is rather an ongoing process directed to new understandings and discoveries, often replacing the old ones [122]. For example, some scientific facts previously accepted by the scientific community as a “consensus” were completely changed in years due to open research, scientific curiosity and freedom in scientific activities. Changes of paradigms or theories occur within an atmosphere open to scientific debate and may often last for years. For example, the role of RNA in the evolution of life has been also heavily challenged in recent years. The presented review is therefore a contribution to a better understanding of the published data covering the topic of COVID-19 without pre-selection of available information.

## Figures and Tables

**Table 2:** Current developmental therapy options for COVID-19 patients. Most of them are in early stages of research and need further clinical studies and validation. The FDA approved drug Ramdesivir was rejected on 19 November 2020 by the World Health Organization due to lack of evidence of its benefits.

Treatment	Description	Recommended Tested Dosage *	References
Chloroquine and Hydroxychloroquine (HCQ) in combination with azithromycin (AZ)	Antimalaric drugs with antiviral characteristics in combination with antibiotics.	200 mg of oral HCQ, three times daily for ten days and 500 mg of oral AZ on day one followed by 250 mg daily for the next four days	Meo et al., 2020 [88] Touret et al., 2020 [89] Lagier et al., 2020 [90].
Regeneron mAb antibody cocktail (REGN-COV2) containing casirivimab (REGN10933) and imdevimab (REGN10987) at equal doses	Neutralizing antibody cocktail containing two SARS-CoV-2 noncompeting, neutralizing human IgG1 antibodies that target the receptor-binding domain of the SARS-CoV-2 spike protein.	2.4 g (low dose), or 8.0 g (high dose)	Weinreich et al., 2020 [91]
Convalescent plasma	Passive immunotherapy with plasma derived from patients convalescent from SARS-CoV-2 infection.	Inconclusive data reported so far	Accorsi et al., 2020 [92] Wood et al., 2021 [93]
Monoclonal antibody treatment bamlanivimab (LY-CoV555)	IgG1 binds to the receptor binding domain of the spike protein SARS-CoV-2, mainly used for the treatment of mild-to-moderate COVID-19 in adult and pediatric patients.	700-milligram dose to 7000-milligram dose yielded similar response	Mahase et al., 2020 [94] Chen et al., 2020 [95]
Interferons (IFNs): interferon beta-1b, Interferon-α2b, lambda interferon, interferon lambda	Mediators of rapid, innate antiviral protection, as prophylactic measure and for early phases of disease.	Different set-ups; i.e., 44-μg/mL (12 million IU/mL) dose of interferon β-1a subcutaneously injected three times weekly for two consecutive weeks	Shalhoub et al., 2020 [96] Zhou et al., 2020 [97] Andreakos et al., 2020 [98] Prokunina et al., 2020 [99] Davoudi-Monfared et al., 2020 [100]
Corticosteroids	Low-to-moderate doses of dexamethasone lower the mortality rate in severe forms of COVID-19, not recommended in patients with mild symptoms. Early, low-dose and short-term application of methylprednisolone correlated with better clinical outcomes in severe patients with COVID-19 pneumonia.	Dexamethasone at 6 mg once daily for up to 10 days; other corticosteroids daily dose equivalencies to dexamethasone 6 mg are: Prednisone 40 mg; Methylprednisolone 32 mg; Hydrocortisone 160 mg	Ahmed et al., 2020 [101] Wang et al., 2020 [102] RECOVERY Collaborative Group, 2020 [73]
Ivermectin	FDA approved antiparasitic drug with activity against COVID-19.	Inconclusive data: 0.15 mg/kg–0.2 mg/kg body weight as a single dosage or 12 mg once daily for 5 days	Pandey et al., 2020 [77] Ahmed et al., 2021 [103] Rajter et al., 2021 [104]
Cytokine inhibitors, i.e., Baricitinib	Halt cytokine storm in COVID-19 patients, might improve survival of the COVID-19 patients.	4 mg/day/for two weeks	Cantini et al., 2020 [105]
Cytosorb	Blood filtration system that filter cytokines from the COVID-19 patients’ blood, reduces cytokine storms.	Therapy via a shaldon catheter for 3–7 days and with filter exchange every 24 h	Stockmann et al., 2020 [106]
Mesenchymal stem cell therapy	Prevent the cytokine storm by the activated immune system along with reparative properties of tissues.	No approved MSC-based approaches for the prevention and/or treatment of COVID-19 patients	Golchin et al., 2020 [107] Rajarshi et al., 2020 [108]
Ventilators and other respiratory support devices	Essential tool for deadly respiratory illness.	Different strategies are applied according to the patient’s state and oxygen saturation values.	Rabec et al., 2020 [109] Mawer et al., 2020 [110] Dondorp et al., 2020 [111]
Anticoagulants, i.e., heparin	Thromboprophylaxis, prevent blood coagulation.	Higher dosages than prophylactic ones showed better survival rates	Connors et al., 2020 [112] Martinelli et al., 2021 [113]
Vitamins and mineral supplements	Boosts the immune system, prevents virus spread and reduces the disease progressing to severe stages.	Inconclusive data	Bae et al., 2020 [114] Bilezikian et al., 2020 [115]

* Recommended tested dosages provided in the table are not immediately transferable in the clinical practice as they stay for specific clinical setups described in the corresponding reference(s) within the table.

## Data Availability

This work did not report any data.

## Abbreviations

ACE2	Angiotensin converting enzyme 2
APE	Acute pulmonary emboli
BCG	Bacillus Calmette-Guérin
BiB	Beijing Institute of Biotechnology
CLIA	Chemiluminescence enzyme immunoassays
COVID-19	Coronavirus disease 2019
DVT	Deep venous thrombosis
ELISA	Enzyme-linked immunosorbent assays
LFIA	Lateral flow immunoassays
LV	Lentivirus vector
MERS	Middle East Syndrom Virus
ORFs	Open reding frames
RBD	Receptor-binding domain
RdRp	RNA-dependent RNA polymerase
RT-PCR	Real-time polymerase chain reaction
SARS-CoV-2	Severe Acute Respiratory Syndrome-Coronavirus 2
SARS-CoV-rS	Covid-19 from Symvivo, recombinant S protein
SMENP	Expressing SARS-COV-2 minigene
WHO	World Health Organization
